# Editorial: Delivery systems in biologics-based therapeutics

**DOI:** 10.3389/fbioe.2023.1274210

**Published:** 2023-08-18

**Authors:** Anjaneyulu Dirisala, Junjie Li, Daniel Gonzalez-Carter, Zheng Wang

**Affiliations:** ^1^ Innovation Center of Nanomedicine, Kawasaki Institute of Industrial Promotion, Kawasaki, Japan; ^2^ Institute for Materials Chemistry and Engineering, Kyushu University, Fukuoka, Japan; ^3^ Molecular Bionics Laboratory, Institute for Bioengineering of Catalonia (IBEC), The Barcelona Institute of Science and Technology (BIST), Barcelona, Spain; ^4^ Key Laboratory of Nano BioInterface, Division of Nanobiomedicine, Suzhou Institute of Nano-Tech and Nano-Bionics, Chinese Academy of Sciences, Suzhou, China

**Keywords:** biologics, nanocarriers, targeting, nucleic acid medicines, protein drugs

The “magic bullet” concept of Paul Ehrlich envisioned site-specific therapies, which opens the door for the design of drugs that selectively attack pathogens and diseased tissue without severe toxicity on healthy tissues (Strebhardt and Ullrich, 2008). For example, antibodies are hailed as prototypical magic bullets because of their inherent specificity against invading pathogens with fewer adverse effects. This idea has motivated scientists of various fields to engineer magic bullet-like drugs for over a century. Biologics are medicines derived from natural sources—humans, plants, animals, or microbes—and may be produced by recombinant DNA technology or other biotechnological methods. Biologics include four classes—sugars, peptide-backbone drugs, nucleotide-backbone medicines, and cell-based therapeutics such as organelles, live cells, and tissues—or combinations of these materials. Approximately 30% of all drugs approved by the US Food and Drug Administration (FDA) in 2015–2018 were biologics; among these biologics, more than 90% were monoclonal antibody-based biologics (Anselmo et al., 2019).

Delivering a therapeutically optimal dose of biologics to the disease site with minimal distribution to healthy tissue would be an advanced treatment modality with high translational promise. Such magic bullet-like targeting of biologics will drastically reduce the required therapeutic dose and minimize toxicity or immunogenicity by avoiding off-target effects. However, each class of biologics often faces unique delivery problems. For example, nucleotide- and peptide-backbone biologics are susceptible to degradation by intravascular nucleases and proteases, respectively, which shortens their half-lives (Uchida and Kataoka, 2019). To solve these challenges, nanoscale delivery systems are often employed to protect the therapeutic cargo from the harsh biological milieu, thereby prolonging their half-lives in the bloodstream to reach the pathological site, enabling release from endolysosomes, effectively trafficking to their intracellular site of action, and reducing off-target side effects.

This Research Topic entitled “*Delivery systems in biologics-based therapeutics*” presents four selected peer-reviewed articles (three reviews and one original research article) that cover the rapidly evolving Research Topic of biologics and their delivery.

Nano-delivery systems hold tremendous potential to be magic bullets. However, maximizing the beneficial biodistribution of nanoparticles to the diseased tissue with minimal off-target biodistribution to other organs to significantly improve the efficacy-to-toxicity ratio still needs to catch up to the great expectations of the magic bullet concept. Towards the design of such site-specific therapies, Cheng et al. provides a review article entitled “*Advances in nanomaterial-based targeted drug delivery systems*,” offering a glimpse of the anatomical and (patho)physiological barriers that hinder tissue and cell-specific drug delivery. Local administrations, for example, intratumoral injection into superficial tumors such as melanoma, offer the simplest means of targeting. However, local therapies are not always clinically feasible when the disease sites are deep and hard to reach. Among systemic administration routes (intravascular, oral, and subcutaneous), intravascular administration offers a wide range of accessibility to various organs and cells through the systemic circulation. However, inside the blood circulation, nanoparticles are subjected to unintended sequestration (both binding to the cell surface and internalization) by the reticuloendothelial system (RES) cells, particularly liver scavenger wall (resident macrophagic Kupffer cells and sinusoidal endothelial cells) (Wen et al., 2023). The endothelial lining of the blood vessels prevents extravasation (penetration) into the target tissue. After successful extravasation, intracellular targeted biologics must enter the cells through endocytosis. Endocytosed nanoparticles must escape from the endosomal compartments to reach their intracellular location of action; for example, messenger (m)RNA must reach the cytosol, whereas plasmid DNA must enter the nucleus. Among these barriers, the liver RES forms the most significant barrier to the clinical translation of nanoparticles because it impedes the delivery of a therapeutically effective dose to the diseased tissue and often raises toxicity concerns. The authors presented recent advancements in different targeting strategies employed by nanosized delivery systems in intravascular (e.g., inflamed endothelium) and extravascular diseases (e.g., cancer). The authors extend their discussions to clinically accepted administration routes other than intravenous injections, such as oral, topical, and inhalation. Noteworthily, these routes of administration eliminate the use of needles, the risk of needle-stick injuries, and the requirements for trained healthcare personnel.

Accurate detection, diagnosis, treatment, and prevention of infectious diseases, including the recent coronavirus disease 2019 (COVID-19) outbreak, have been a significant burden for humankind. Considering the high incidence of infections by bacteria and viruses, and their resistance to conventional antimicrobials, infectious diseases may become the most severe global threat, which strongly urges the development of novel antimicrobial nanomaterials. To this end, Chen et al., and their team wrote a comprehensive review on “*The role of nanotechnology-based approaches for clinical infectious diseases and public health*.” In this review article, the authors initially focused on the current burden of infectious diseases on healthcare systems, mainly on bacterial infections and the recent viral pandemic of COVID-19 caused by severe acute respiratory syndrome coronavirus 2 (SARS-CoV-2). Later, the team highlighted how the nanotechnological approaches aided the existing diagnosis and treatment modalities against emerging infectious diseases. The authors also discussed the healing and treating activities of metallic and non-metallic nanoparticles against bacterial infection-accompanied wounds.

One of the critical features of peptide-backbone-based biologics (ranging from small peptides to large monoclonal antibodies) towards magic bullet-like properties is the high specificity and affinity to their targets, as these features generally possess favorable biodistribution profile with minimal off-target effects. Hence, understanding the molecular mechanisms underlying hierarchic binding and vast different affinities between immunoglobulin G (IgG) antibodies and their FcγRs (fragment, crystallizable (Fc), gamma receptors) is critical for the engineering of antibodies. The recent crystal structures of the FcγRI-Fc complex showed that high-affinity FcγRI binds to IgG in a similar mode to those of the low-affinity FcγR, resulting in a controversial structural discrepancy that led to two contradicting structural mechanisms for the high-affinity binding between FcγRI and IgG. To resolve this controversy, Lu et al. confirmed that the characteristic FG loop-Fc interaction is critical to the high-affinity binding in their original article “*Fc*γ*RI FG-loop functions as a pH-sensitive switch for IgG binding and release*.” The structural and mutational analyses revealed that high-affinity IgG binding of FcγRI is a pH-sensitive mechanism, where the extracellular D2 domain FG loop of FcγRI functioned as a pH-sensing switch for IgG binding. In addition, the live cell imaging studies revealed that FcγRI-mediated uptake of immune complexes prepared from bovine serum albumin (BSA) and anti-BSA IgG antibody is a pH-sensitive temporal-spatial antibody-antigen uptake and release.

Besides peptide-backbone biologics, there is an increasing interest in the delivery of nucleotide-backbone therapeutics, which can fine-tune cellular responses. In disease conditions, the “in-body drug factories (DNA and mRNA)” fail, resulting in decreased or anomalous protein expression. Theoretically, we can treat diseases by exogenous delivery of these nucleic acid drugs to correct the protein expression levels. Among all nucleic acid drugs, mRNA therapeutics have garnered considerable attention as versatile drugs for protein replacement therapy and preventive vaccines against infectious diseases because of their ability to enable efficient and controlled protein expression without the risk of random integration into host genomes (Hajj and Whitehead, 2017; Chaudhary et al., 2021). Indeed, the extraordinary transformative potential of mRNA therapeutics to clinics have already been witnessed by the recent approval of two mRNA vaccines against COVID-19: Spikevax^®^ (Moderna) and Comirnaty^®^ (Pfizer/BioNTech), which led mRNA therapeutics to the forefront of medicine. Cardiovascular disorders remain the leading cause of mortality and morbidity worldwide despite advances in curative and preventive medicine. mRNA therapy is a promising approach for treating cardiovascular diseases because mRNA circumvents the critical difficulties of conventional protein- and DNA-based gene therapy. Towards the rapid development of mRNA delivery to the heart, Wang et al. reported a review article on “*mRNA therapy for myocardial infarction: A review of targets and delivery vehicles.*” In the first half of their article, the authors compiled three potential targets for cardiac mRNA therapy, increasing cardiomyocyte proliferation, reducing fibrosis, and promoting angiogenesis. Later, the authors provided an expert view of the delivery challenges encountered by mRNA drugs and their delivery vehicles, ranging from viral carriers to nonviral delivery systems, including lipid nanoparticles (LNPs) and polymeric vehicles. Although the current FDA-approved LNPs are clinically-matured technologies, the pro-inflammatory concerns of ionizable cationic lipids (Ndeupen et al., 2021) limit their applications other than as vaccines. Furthermore, the incidence of post-injection side effects in the heart, such as myopericarditis and endocarditis (Li et al., 2021), can be a hurdle to the widespread applications of LNPs. To fully realize the magic bullet potential of LNPs, site-selective mRNA translation (i.e., decoding mRNA to build a chain of amino acids) exclusively at the injection site is highly preferable because such localized protein expression drastically decreases systemic toxicity and immunogenicity concerns. However, current FDA-approved mRNA-loaded LNP-based vaccines fail to achieve ‘on-target’ protein expression in immunologically-active tissues due to their high propensity for liver accumulation even after local intramuscular administration (Pardi et al., 2015; Lindsay et al., 2019), which limits their magic bullet-like properties. Disappointingly, organ biodistribution studies confirmed that a significant portion of injected mRNA-LNP migrates to the liver despite its intended muscle localization (injection site) (Lindsay et al., 2019). This high hepatic off-targeting was reported to induce adverse effects in the liver, such as unwanted necrosis of liver cells and T cell–dominant immune-mediated hepatitis, possibly due to the undesired expression of antigens in the liver (Chen et al., 2022). Furthermore, such hepatic off-targeting limits the application potential of mRNA to people with predisposed inflammation and immune-challenged conditions such as chronic or acute systemic infections and exacerbates inflammation (Parhiz et al., 2022). Hence, reducing the liver uptake and rerouting mRNA vaccines to immunologically-active lymphoid organs will benefit widespread vaccine applications. Considering this Research Topic, the current research paradigm is heading in several directions. One direction intensively focuses on pursuing other delivery carriers, such as (lipo)polyplexes (Thalmayr et al., 2023) and polyplex micelles (Uchida and Kataoka, 2019). The other direction focuses on developing liver-RES blockade strategies such as transient and selective *in situ* stealth coating of liver scavenger sinusoidal wall cells using two-armed poly(ethylene glycol)–conjugated oligo(l-peptide) to prevent the unwanted liver uptake of nanoparticles and thereby rerouting the nanoparticles to target organs (Dirisala et al., 2020). Noteworthily, progressive biliary excretion of stealth coating agents after its intended medical use overcomes the regulatory concerns associated with chronic accumulation and patient safety. The another direction focuses on developing nanoparticles with selective organ targeting (SORT) properties with the advent of endogenous targeting mechanisms–wherein the molecular composition of the nanoparticle is rationally designed to bind with distinct subsets of biomolecules in the blood upon injection to bypass the liver accumulation and target specific organs (Dilliard et al., 2021). For example, LNPs prepared from permanently cationic 1,2-dioleoyl-3-trimethylammonium-propane and negatively charged 1,2-dioleoyl-*sn*-glycero-3-phosphate are acting as SORT molecules to enable enriched adsorption of vitronectin and *β*2-glycoprotein I for preferential uptake by lung and spleen, respectively.

The present Research Topic provides comprehensive reviews of the design criteria of various nanosized delivery systems for the effective delivery of biologics. Each article examines possible strategies to overcome the existing limitations of biologics delivery. We perceived a scientific consensus that future studies should be devoted to improving the targeting efficiency of biologics to diseased tissue without adverse effects in healthy tissue (i.e., magic bullet-like targeting). We believe that the Articles of this Research Topic will inspire researchers to engineer next-generation delivery systems to harness the full therapeutic and diagnostic potential of biologics, thereby filling the gaps in the bench-to-bedside clinical translation process of biologics-based nanotherapeutics.
